# In-Kennel Behavior Predicts Length of Stay in Shelter Dogs

**DOI:** 10.1371/journal.pone.0114319

**Published:** 2014-12-31

**Authors:** Alexandra Protopopova, Lindsay Renee Mehrkam, May Meredith Boggess, Clive David Lawrence Wynne

**Affiliations:** 1 Department of Psychology, University of Florida, Room 114, Psychology Bldg, P.O. Box 112250, Gainesville, FL, 32611-2250, United States of America; 2 School of Mathematics and Statistical Sciences, Arizona State University, Tempe, AZ, 85281, United States of America; 3 Faculty of Health and Medicine, University of Newcastle, Callaghan, NSW, 2308, Australia; 4 Department of Psychology, Arizona State University, Tempe, AZ, 85281, United States of America; University of Florida, United States of America

## Abstract

Previous empirical evaluations of training programs aimed at improving dog adoption rates assume that dogs exhibiting certain behaviors are more adoptable. However, no systematic data are available to indicate that the spontaneous behavior of shelter dogs has an effect on adopter preference. The aim of the present study was to determine whether any behaviors that dogs exhibit spontaneously in the presence of potential adopters were associated with the dogs' length of stay in the shelter. A sample of 289 dogs was videotaped for 1 min daily throughout their stay at a county shelter. To account for differences in adopter behavior, experimenters varied from solitary passive observers to pairs of interactive observers. Dogs behaved more attentively to active observers. To account for adopter preference for morphology, dogs were divided into “morphologically preferred” and “non-preferred” groups. Morphologically preferred dogs were small, long coated, ratters, herders, and lap dogs. No theoretically significant differences in behavior were observed between the two different dog morphologies. When accounting for morphological preference, three behaviors were found to have a significant effect on length of stay in all dogs: leaning or rubbing on the enclosure wall (increased median length of stay by 30 days), facing away from the front of the enclosure (increased by 15 days), and standing (increased by 7 days). When combinations of behaviors were assessed, back and forth motion was found to predict a longer stay (increased by 24 days). No consistent behavioral changes were observed due to time spent at the shelter. These findings will allow shelters to focus behavioral modification efforts only on behaviors likely to influence adopters' choices.

## Introduction

The American Society for the Prevention of Cruelty to Animals [1] estimates that 5 to 7 million pets are admitted to shelters each year, with approximately 60% of admitted dogs ultimately euthanized [1]. Many shelters are managed on scarce private donations or limited public funds, resulting in impoverished living conditions for the animals housed [2].

In order to improve living conditions and decrease euthanasia rates, animal welfare organizations are advocating the use of behavior modification programs to improve dog behavior while at the shelter [3]. Despite the growing acceptance of such programs, few studies have investigated the effects of behavioral training on improving adoption success in shelter dogs. Furthermore, the outcomes of those studies that have evaluated the influence of behavior modification on adoption rates have reported mixed results. Braun [4] reported that in the few years in which a volunteer-run training program was implemented at a shelter, the number of dogs that had a prolonged length of stay decreased. In a more systematic study, Luescher and Medlock [5] found that obedience training had a positive influence on adoption rates. The intervention consisted of training a variety of different behaviors, such as walking on a head halter, sitting on cue, not jumping on people, not barking in the kennel, and staying in the front portion of the kennel. The multiplicity of behaviors trained makes it difficult to pinpoint the exact behaviors that were necessary and sufficient to increase adoptions. Protopopova et al. [6] evaluated whether training shelter dogs on a social behavior, specifically gazing into the eyes of adopters, increased adoption rates. Although the experimental manipulation did increase gazing toward experimenters, this did not significantly increase adoption rates. Similarly, Herron et al. [7] found that training the dogs on a multitude of in-kennel behaviors increased the occurrence of some of those behaviors, but had no impact on length of stay. These few published studies do not provide clear conclusions on the effects of training. Furthermore, these studies do not identify the exact training regimen that may be sufficient or necessary to improve adoptions.

The authors of the studies described above developed their interventions based on assumptions as to which behaviors are attractive to adopters. Recently, a few studies have empirically evaluated which, if any, spontaneous behaviors of dogs in shelters actually correlate with improved adoption. Protopopova and Wynne [8] found that dogs which spend less time ignoring play initiations by adopters and more time lying in proximity to adopters have a higher likelihood of adoption; however, the authors only assessed the dog's behavior once it was taken out of the kennel. Waller et al. [9] found that dogs who exhibited juvenile-like behaviors in the kennel, namely eyebrow lifting, had a shorter length of stay. Whereas this finding is intriguing, it does not permit clear guidelines for behavioral training to improve adoption. To complicate things further, dogs' behavior may change due to time alone. For example, Stephen and Ledger [10] found that the dogs in their population spent more time hiding out of view and were less responsive to external social stimuli with time spent at the shelter; whereas Beerda et al. [11] and Hetts et al. [12] found an increase in locomotion in laboratory dogs with time.

Several studies have used relatively indirect measures in attempts to determine what makes a dog attractive to adopters. For example, Wells and Hepper [13] distributed questionnaires to 100 randomly chosen members of the general public in which they had to choose between two photographs of dogs that differed in one specific way. Using this choice task, the results suggests that people prefer dogs that are labeled as “unwanted” versus “stray,” “have a clean cage” versus “dirty cage,” “don′t bark” versus “bark,” “are in front of the cage” versus “back of the cage,” and “have a ball” versus “do not have a ball.” Wells and Hepper also found that when asked what determines a dog's attractiveness for adoption, participants answered that temperament is the most important factor, followed by size, sex, appearance, and age [13]. More recently, Weiss et al. [14] asked adopters, at the time of their adoption of a pet, what behaviors their newly adopted dog engaged in during the first meeting. Adopters reported that dogs approached and greeted, licked, jumped on them, and wagged their tails during the meeting. The authors suggest that these behaviors might have influenced adopters' choices. Whereas these studies provide a good starting point for further research, the indirect methods of measurement of adopter preference through surveys limit utility of these studies in developing effective interventions.

Other studies have correlated morphological and background factors of dogs with adoption rates. Adopters prefer light over dark colored dogs [13], [15], owner surrenders over strays [6], [13], long-haired over short-haired dogs [13], young over old dogs (see discussion by Brown et al., [16]), neutered over intact dogs [15], [17], and toy breeds over other breeds [6] (also see discussion by Brown et al., [16]). In fact, Protopopova et al. [6] found that potential adopters rated photos of adopted dogs as more physically attractive than dogs that were euthanized, suggesting that morphology plays a significant role in the choice of dog. In addition, Weiss et al. [14] reported that adopters claimed appearance to be the single most important reason to adopt a dog. Therefore, morphological traits must be taken into account when assessing the effects of behavior on adopter selection. Whereas many factors, such as breed, color, age, coat length, neuter status, and mode of intake, seem to influence adoption rate, no clear evidence of behavioral factors have been reported that correlate with adoption rates.

The primary aims of the present study were to assess whether behavior exhibited by dogs inside the kennel influenced length of stay in the shelter and whether the morphology of the dogs mediated this influence. To achieve these aims, we first determined which morphological variables influenced length of stay in our population. Second, in order to avoid confounding the effects of behavior on length of stay with changes in behavior due to time itself, we evaluated the effect of time alone on in-kennel behavior of shelter dogs. Third, we evaluated the effect of the type of human attention on in-kennel behavior of shelter dogs. Finally, we assessed which behaviors influenced length of stay, while accounting for the variables mentioned above. Together, these findings have the potential to contribute to a more systematic approach to the development of targeted interventions aimed at increasing adoption rates in shelter dogs.

## Materials and Methods

### Animals and housing

Dogs (n = 349), which were available for adoption at the Alachua County Animal Services (ACAS) in Gainesville, FL, from the beginning of May through December 2012, served as subjects for this study. ACAS is an open-admission county animal shelter that functions both as animal control and adoption facility. Adoptable dogs comprised of seized (including stray) and owner-surrendered dogs that passed an informal behavioral assessment and veterinary examination conducted by shelter staff.

Dogs were housed in two rows of adjacent kennels with cement walkways in front and back. With the exception of litters of puppies and a few pairs of dogs, the animals were individually housed in 1.0 m×4.6 m×2.1 m kennels with two-thirds of the pen outdoors and one-third inside. The dogs could be viewed by the public from the outside walkway. All kennels had cement floors and 1.2 m tall cement walls that were connected to the ceiling of the kennel with a chain-link fence. Each kennel contained a water dish, a food dish, and a Kuranda bed (Kuranda USA, Annapolis, MD, USA) in the inside portion of the kennel, out of the sight of the public. Staff fed the dogs and cleaned kennels daily before 9:30h. Volunteers at the shelter occasionally (approximately twice per week) exercised, trained, and played with the dogs outside the kennels but on the shelter premises.

A cage card was attached to each kennel that contained the dog's name, identification number, sex, age, breed (as determined by shelter staff), mode of intake (surrendered by the owner, found as a stray, or confiscated by animal control), and, infrequently, a few words on the history of the dog.

Independent rescue organizations selected dogs weekly to be placed into their programs. Dogs were marketed by the shelter staff and volunteers on their website, several national online databases, local news channels, and through a popular online social networking site. Dogs that were perceived as hard-to-adopt by shelter staff based on an extended length of stay at the shelter had a lower adoption fee. No dogs were euthanized due to lack of space during the time of the study.

### Behavioral coding

An ethogram was developed based on preliminary observations of in-kennel behaviors exhibited by dogs at ACAS, as well as from other ethograms previously published in peer-reviewed scientific journals and case studies. This full list of all behaviors and their operational definitions are available in Table 1. In addition to these 42 behaviors, the cleanliness of the dog's kennel was recorded each day; the observer noted whether feces or vomit were present or absent in the kennel during the time of the video recording.

**Table 1:** Ethogram of In-Kennel Behaviors of Shelter Dogs.

Behaviour	Operational Definition
**Body position**	
Front of kennel	Located between front of cage, and up to and including the midpoint of kennel
Back of kennel	Located between back wall of kennel, and up to, but not including, midpoint of kennel.
Lying down	Lying down with limbs either tucked under or placed in front of body
Out of sight	Not visible from the front of the cage, behavior cannot be defined [29]
Sitting	Supported by two extended front legs and two flexed back legs [30]
Standing	Supported upright with all four legs [30]
Belly up	Lying/sitting on ground lifting hind leg, or rolling onto back exposing ventral side
Beg position	Two front paws lifted off the ground simultaneously while the back legs remain flexed.
Cowering	Body in a lowered, crouched position
Play bowing	Lowered anterior and heightened posterior part (standing on hind-legs) [29]
Pawing at door	One front paw makes contact with the cage door
**Face Orientation**	
Facing forward	Head is oriented such that the observer is able to see more than the side profile of face
Facing away	Head is oriented such that the observer is not able to see more than the side profile of face
Gazing	Eye contact with the eyes of the observer
Tilting head	Entire head is quickly oriented laterally and held stationary for at least 1sec
Ears back	Ears folded against sides and/or back of head and having a flattened appearance.
**Tail position**	
Tucking tail	Tail held still and tightly between hind legs, may be curled under genital area or ventral side
Wagging tail	Tail moves perpendicular to the dog's body
**Locomotion**	
Moving forward	Distance between the dog and the observer is decreased
Moving away	Distance between the dog and the observer is increased
Jump on cage	Both front paws make contact with the cage door that does not include lunging
Lunging	Quick diagonal forward motion; may be accompanied by barking, growling or piloerection
Pacing	Repeatedly (>3) locomoting around kennel in fixed route [31]
Chasing tail	Orients towards tail repeatedly (>3) and continuously (adapted from [31]]
**Vocalization**	
Barking	Vocalization of very short duration and low frequency [32]
Growling	Throaty, rumbling vocalization; usually low in pitch
Howling	Prolonged high-amplitude vocalization of varying pitch, lips drawn together while exhaling
Whining	A cyclic vocalization [32]
**Enclosure contact/exploration**	
Leaning on door	Prolonged (>1 sec) contact with the cage door by pushing side of body against the cage door
Licking kennel	Repeatedly chews, licks, and/or bites at the wire of the cage door [12].
Leaning on wall	Prolonged (>1sec) contact with the cage wall by pushing side of body against the cage wall
Sniffing	Muzzle/nose is oriented in a clearly observable direction and motion of nostrils is observed
**Grooming**	
Scratching	Paw makes repeated contact with body/face; head may be angled in direction of moving limb
Licking self	Oral contact with any part of body
Shaking off	Motions body and/or head back and forth repeatedly and rapidly
**Maintenance**	
Yawning	Opens mouth widely and inhales [33]
Stretching	Extending body and one or more front and/or hind-legs while remaining stationary
Panting	Tongue exposed with audible and/or observable breathing
Trembling	Visible shaking while dog is standing still or cowering
Regurgitating	Matter expelled from mouth with jaws open; may be preceded by repeated abdominal heaving
Eliminating	A hind-leg lifted or is squatting and urinates/defecates [29]
Coprophagy	Feeding on own/other dogs' feces [29]

### Data collection

Behaviors for each dog were recorded daily using a Kodak PlaySport Zx3 video camera using the WVGA mode at 30 fps (Kodak Company, Rochester, NY, USA). On the days that the shelter was open (Tuesday through Saturday), an observer approached the cage as an adopter would, and stood facing the dog's kennel for one minute recording with the video camera the dog's spontaneous behavior. The duration for which the dogs' behaviors were recorded was chosen based on previous research which showed that adopters only look at an individual dog for 20-70 s [18]. After the minute had elapsed, the observer moved to the next kennel and repeated this procedure until every dog available for adoption in the shelter had been recorded. On two of the five days in the week, two observers approached the kennel. One observer filmed the dog as previously described and the other crouched down and gently spoke with the dog from outside the kennel. This modification was incorporated in order to imitate the typical behavior and number of adopters when visiting the shelter. The dogs were filmed in this manner between 10:00h and noon. The dogs were not filmed if they were locked in the inside portion of the kennel or not present in the kennel. Data collection did not occur on days on which it rained heavily. Eleven female observers collected data on the subjects in order to limit possible habituation effects to individual people.

Videos were coded in 5 s intervals for behaviors listed in Table 1. An interval was scored as positive for a behavior if it was observed at any point during that interval. All coders were undergraduate students trained to 90% agreement on practice videos prior to data coding. In order to assess inter-observer reliability, 349 videos (19.2%) were coded by two observers and agreement was scored when the two observers agreed on the occurrence of a behavior in each 5 s interval. For each video, the scores were averaged to give a proportion of a minute (i.e. number of intervals behavior observed divided by 12) in which each behavior was observed; this proportion was then multiplied by 100 to derive the percentage.

Morphological data for each dog was also recorded from the cage card: sex (male or female, all were spayed/neutered at the time of final adoption), age (puppy – less than 4 months, adult – between 4 months and 7 years, or senior – over 7 years), height (small – 0.35 m; medium – between 0.35 m and 0.60 m; and large – over 0.60 m), mode of intake (surrender, stray, or confiscated), coat length (short or long), and breed. The breeds were grouped together into seven types: Ratters, Fighting, Hound, Working, Herding, Sporting, and Lap. These categories were modified from Lepper et al. [15] and further explained in Protopopova et al. [6]. Previous research had suggested that size, coat length, age, and breed influenced adoption likelihood: possibly preferred were dogs with small size, long coat, light color, aged less than 4 months, owner surrendered, or of certain breed types [6], [13], [15], [16].

The outcome and length of stay for the dogs were obtained from the shelter records at the conclusion of the study. Possible outcomes were adoption, placement into rescue organization, or euthanasia (for medical reasons). Euthanized dogs were not included in the study as the length of stay was not indicative of their desirability for adoption.

### Ethics statement

All procedures were approved by the University of Florida Institutional Animal Care and Use Committee (#201207467).

### Data analysis

All data manipulation and statistical analysis was carried out in Stata MP13.1 (Stata LP, College Station, TX). Raw data and all codes generated in Stata for data analysis can be accessed in DRYAD (datadryad.org; doi:10.5061/dryad.5n7p9).

#### Morphologically preferred dogs

Since morphology has previously been identified as an important factor in adoption choices, our first aim was to parse out these effects from those of behavior. Parametric survival models were used to determine the effect on length of stay of all morphological variables and the Gompertz distribution was identified, by the lowest Akaike information Criterion (AIC), to yield the best fitting models.

#### Behavior by morphology

We would not expect all dogs to behave in the same way, which may lead to a confounding of some characteristic, such as breed type, with behavior. Thus, it is important to determine whether there are differences in behavior by morphology. To estimate the variability of behavior by morphology, we fit a logistic regression model, with a random effect for dog, to each behavior dichotomized to “1” if the dog had performed it at all during that video and “0” otherwise. Breed type, size, and length of coat were used as explanatory variables.

#### Morphological and behavioral descriptive summary

For descriptive purposes only, dogs were partitioned into four groups, formed according to a split on length of stay (shorter or longer than 30 days) and morphological preference group (preferred or non-preferred morphology). Table 2 summarizes morphological information by preference group and length of stay. The median length of stay was 14 days (IQR 6–32, range: 1–71); however, because of the bimodal distribution of the data (many morphologically non-preferred dogs stayed for longer than 14 days, while preferred dogs stayed for less than 14 days), we divided the length of stay at 30 days instead of the median to ensure an approximately equal number of dogs in the “short” and the “long” stay category.

**Table 2:** Summary of Morphological Variables.

		Preferred		Non-preferred	
	Total	Stay<30 days	Stay≥30 days	Stay<30 days	Stay≥30 days
Number of dogs	289	107	14	101	67
**Median age (months)**	8	4	3	9	12
**Male**	131 (45.3%)	46 (43.0%)	6 (42.9%)	48 (47.5%)	31 (46.3%)
**Long coat** a	20 (6.9%)	20 (18.7%)	0 (0.0%)	0 (0%)	0 (0%)
**Dog size**					
Small a	92 (31.8%)	81 (75.7%)	11 (78.6%)	0 (0%)	0 (0%)
Medium	181 (62.6%)	24 (22.4%)	3 (21.4%)	89 (88.1%)	65 (97.0%)
Large	16 (5.5%)	2 (1.9%)	0 (0%)	12 (11.9%)	2 (3.0%)
**Breed type**					
Ratter a	17 (5.9%)	15 (14.0%)	2 (14.3%)	0 (0%)	0 (0%)
Fighting	83 (28.7%)	17 (15.9%)	2 (14.3%)	39 (38.6%)	25 (37.3%)
Hound	47 (16.3%)	9 (8.4%)	0 (0%)	20 (19.8%)	18 (26.9%)
Working	25 (8.7%)	3 (2.8%)	1 (7.1%)	13 (12.9%)	8 (11.9%)
Herding a	27 (9.3%)	26 (24.3%)	1 (7.1%)	0 (0%)	0 (0%)
Sporting	75 (26.0%)	22 (20.6%)	8 (57.1%)	29 (28.7%)	16 (23.9%)
Lap a	15 (5.2%)	15 (14.0%)	0 (0%)	0 (0%)	0 (0%)
**Color**					
White	13 (4.5%)	4 (3.7%)	1 (7.1%)	4 (4.0%)	4 (6.0%)
Gray	6 (2.1%)	0 (0%)	0 (0%)	5 (5.0%)	1 (1.5%)
Tan	55 (19.0%)	27 (25.2%)	3 (21.4%)	20 (19.8%)	5 (7.5%)
Red	33 (11.4%)	10 (9.3%)	2 (14.3%)	14 (13.9%)	7 (10.4%)
Black	31 (10.7%)	12 (11.2%)	2 (14.3%)	12 (11.9%)	5 (7.5%)
Red and white	30 (10.4%)	8 (7.5%)	0 (0%)	13 (12.9%)	9 (13.4%)
Black and white	46 (15.9%)	16 (15.0%)	6 (42.9%)	12 (11.9%)	12 (17.9%)
Black and tan	21 (7.3%)	9 (8.4%)	0 (0%)	6 (5.9%)	6 (9.0%)
Tri-color	14 (4.8%)	4 (3.7%)	0 (0%)	6 (5.9%)	4 (6.0%)
Brindle	34 (11.8%)	13 (12.1%)	0 (0%)	7 (6.9%)	14 (20.9%)
Merle	6 (2.1%)	4 (3.7%)	0 (0%)	2 (2.0%)	0 (0%)
**Intake Mode**					
Stray	219 (75.8%)	77 (72.0%)	9 (64.3%)	81 (80.2%)	52 (77.6%)
Owner surrender	67 (23.2%)	28 (26.2%)	5 (35.7%)	20 (19.8%)	14 (20.9%)
Confiscated	3 (1.0%)	2 (1.9%)	0 (0%)	0 (0%)	1 (1.5%)

Morphologic summary by length of stay and preference group for N = 289 dogs.

a Variables that comprise the morphologically preferred group (long coat, small size, or ratter, herder, or lap dog).

#### Behavior change with attention

For 533 (30%) videos, an additional person bent down and paid attention to the dog through the cage door. It is possible that some dogs' behavior was affected by this additional attention, and if so, this may have impacted our analysis of the impact of behavior on length of stay. Therefore, for each behavior in Table 1, a logistic regression model with a random effect for dog, was fit to estimate the association of human attention with dog behavior, whilst controlling for morphological preferences. A logistic model estimates an odds ratio (OR) for each explanatory variable and probability of the behavior at each level of the explanatory variable. An odds ratio greater than one indicates the behavior increased with attention and an odds ratio less than one indicates the behavior decreased.

#### Behavior change due to time alone

It is possible that some dogs' behavior changed during their shelter stay. If so, this could give us the impression that that behavior caused the longer stay rather than resulting from a longer stay. To assess the impact of time in shelter on dog behavior, we fit a logistic regression model for each behavior in Table 1, for each dog with more than 5 observations, whilst controlling for human attention.

#### Effect of behavior on length of stay

Parametric survival models with a Gompertz distribution were used to assess the effect of behavior on length of stay while controlling for three morphological variables: length of coat, size and dog breed type (ratter, herder or lap dog versus all others).

Parametric survival models quantify effects in two ways: hazard ratios (HR) and estimated median survival time. An HR greater than one indicates that an increase in that variable is related to an increased hazard, that is, a reduction in survival time. For this study, survival time was length of stay, an HR greater than one indicated that an increase in that variable was related to a shorter length of stay. On the other hand, an HR less than one indicated that an increase in that variable was related to a greater length of stay. A parametric survival model can be used to estimate the median survival time, in much the same way as a linear regression model can be used to estimate the mean of a quantity. So another way to describe the effect of a variable on survival time is to calculate the median survival time at one value of the variable and compare that to the median survival time at a different value of the same variable. For example, we could compare the median survival time of a dog performing a behavior to one that does not perform that behavior. Given that we have morphological variables in the model as well, we could also, for example, compare the median survival time of a small dog to the median survival time of a larger dog.

## Results

A total of 349 adoptable dogs were housed for some period of time at ACAS during the study period, of which 22 were euthanized for medical reasons. For 12 dogs, no outcome information was available in shelter records (due to human error in recording the identifying numbers). Furthermore, 26 additional dogs were adopted during a large adoption event where adoption decisions were made in an atypical environment, and, thus, these dogs were not included in the final analyses. This left 289 dogs available for analyses with a total of 1,766 video observations. One behavior, ears back, had a low interobserver agreement (71.6%), and was therefore removed from the analysis, leaving 41 behavioral variables. The remaining behavioral variables had an average interobserver agreement of 95.7% (range: 80.2–100%).

### Morphologically preferred dogs

Having a long coat was found to significantly shorten length of stay (P<0.001). When size was added to this model, there was no difference between medium and large dogs (P = 0.488), so size was recoded into two categories (small vs medium or large). Small size significantly shortened length of stay (P<0.001).When ratter type breed was added to this model, it too significantly shortened length of stay (P = 0.004). When breed type either ratter or lap dog was added to the model containing long coat and small size, it made a significant contribution (P = 0.001). When breed type either ratter, lap dog or herder was added to the model containing long coat and small size, it was statistically significant (P<0.001). No other breed types had a significant impact on length of stay; thus, breed type was recoded into two groups: ratter, lap dog or herder (preferred breed type) versus all other breed types (non-preferred breed type).

When age under 4 months of age was added to the model, it was not significant (P = 0.546), which is not surprising since 70% of these young dogs were classified as small by shelter staff and 81% of older dogs were classified as medium or large; thus, once small size was included in the model, it was accounting for the effect of being a young dog. When indicators for intake mode (owner surrender, confiscated, or stray) were added to this model, no difference was found (P = 0.550). When adding the color of the dog to the model, only merle was shown to be preferred (P = 0.029). However, there were only six merle dogs in the study, so that together with the lack of significance of any other coloring, dog color was discarded from further consideration for morphological preference. Thus, for the remainder of this analysis, we considered morphologically preferred dogs to be of a small size, long coat, or of preferred breed type (ratter, herder or lap). Twenty dogs (6.9%) had a long coat, 92 dogs (31.8%) were small, 17 (5.9%) ratters, 27 (9.3%) herders, 15 (5.2%) lap dogs, and 74 (25.6%) dogs were under 4 months of age.

### Behavior by morphology

We found that medium to large dogs and dogs with long coats were more likely to be out of sight (P = 0.005, 0.003, respectively), in the back of the kennel (P = 0.019, <0.001, respectively) and facing away from the observer (P = 0.025, 0.019, respectively). They were also more likely to be moving from the back of the kennel towards the observer (P = 0.034, 0.013, respectively), and from the front of the kennel away from the observer (P = 0.008, 0.011, respectively). Additionally, they were more likely to be panting (P = 0.003, <0.001, respectively). Medium to large dogs were more likely to whine (P = 0.017) and stretch (P = 0.004). As a group, ratters, herders and lap dogs are more likely to whine (P = 0.019) and less likely to be lying down (P = 0.042). The only significant effect of age was that dogs under the age of 4 months were more likely to sit (P<0.001).

### Behavioral descriptive summary

Table 3 displays the counts and percentages of behaviors observed at least once during all 1 min video observations. Longer stay dogs appeared more likely to be at the back of the kennel, moving forward and away, standing, or growling. Morphologically preferred dogs appeared to lie down more often and were somewhat more often out of sight. Morphologically non-preferred dogs were more likely to be leaning/rubbing the enclosure wall, barking, whining, or to have feces in the enclosure. Shorter stay dogs appeared to be pacing, whining, or barking somewhat more often if they were morphologically preferred, and sitting, lying down, licking themselves, yawning or panting somewhat more often if they were morphologically not preferred. Whereas these descriptive results can be suggestive of the relative importance of variables under consideration, multivariable models are needed to take into account the effects of more variables simultaneously and enable inferences about behaviors whilst controlling for other factors.

**Table 3:** Descriptive Statistics of All Behaviors.

		Preferred		Non-preferred	
	Total	Stay<30 days	Stay≥30 days	Stay<30 days	Stay≥30 days
Number of days	1,766	287	474	131	874
Position in Kennel					
Front of kennel	1691 (96%)	272 (95%)	124 (95%)	456 (96%)	839 (96%)
Back of kennel	474 (27%)	63 (22%)	33 (25%)	109 (23%)	269 (31%)
Out of sight	349 (20%)	51 (18%)	23 (18%)	78 (16%)	197 (23%)
Face Orientation					
Facing forward	1700 (96%)	273 (95%)	127 (97%)	456 (96%)	844 (97%)
Gazing	1562 (88%)	252 (88%)	114 (87%)	414 (87%)	782 (89%)
Facing away	1098 (62%)	171 (60%)	75 (57%)	282 (59%)	570 (65%)
Tilting head	105 (5.9%)	24 (8.4%)	9 (6.9%)	23 (4.9%)	49 (5.6%)
Locomotion					
Moving forward	542 (31%)	77 (27%)	40 (31%)	118 (25%)	307 (35%)
Moving away	542 (31%)	78 (27%)	41 (31%)	122 (26%)	301 (34%)
Jumping on cage	618 (35%)	105 (37%)	43 (33%)	163 (34%)	307 (35%)
Chasing tail	7 (0.4%)	0 (0%)	0 (0%)	0 (0%)	7 (0.8%)
Lunging	4 (0.2%)	2 (0.7%)	0 (0%)	0 (0%)	2 (0.2%)
Pacing	18 (1.0%)	6 (2.1%)	1 (0.8%)	0 (0%)	11 (1.3%)
Body Position					
Standing	1237 (70%)	184 (64%)	90 (69%)	321 (68%)	642 (73%)
Sitting	874 (49%)	165 (57%)	72 (55%)	239 (50%)	398 (46%)
Lying down	537 (30%)	68 (24%)	47 (36%)	163 (34%)	259 (30%)
Pawing at door	370 (21%)	59 (21%)	19 (15%)	112 (24%)	180 (21%)
Play bowing	120 (6.8%)	16 (5.6%)	7 (5.3%)	38 (8.0%)	59 (6.8%)
Belly up	13 (0.7%)	3 (1.0%)	2 (1.5%)	4 (0.8%)	4 (0.5%)
Beg position	6 (0.3%)	0 (0%)	1 (0.8%)	3 (0.6%)	2 (0.2%)
Cowering	17 (1.0%)	2 (0.7%)	2 (1.5%)	6 (1.3%)	7 (0.8%)
Tail position					
Wagging tail	1393 (79%)	213 (74%)	95 (73%)	372 (78%)	713 (82%)
Tucking tail	7 (0.4%)	2 (0.7%)	0 (0%)	2 (0.4%)	3 (0.3%)
Vocalization					
Barking	614 (35%)	100 (35%)	27 (21%)	159 (34%)	328 (38%)
Whining	440 (25%)	70 (24%)	16 (12%)	115 (24%)	239 (27%)
Growling	61 (3.5%)	4 (1.4%)	5 (3.8%)	12 (2.5%)	40 (4.6%)
Howling	50 (2.8%)	10 (3.5%)	0 (0%)	12 (2.5%)	28 (3.2%)
Enclosure contact/exploration					
Sniffing	811 (46%)	148 (52%)	66 (50%)	188 (40%)	409 (47%)
Lean/rub on wall	287 (16%)	27 (9.4%)	10 (7.6%)	62 (13%)	188 (22%)
Lean/rub on door	39 (2.2%)	4 (1.4%)	1 (0.8%)	12 (2.5%)	22 (2.5%)
Lick/chew kennel	221 (13%)	38 (13%)	14 (11%)	62 (13%)	107 (12%)
Grooming					
Licking self	157 (8.9%)	29 (10%)	14 (11%)	55 (12%)	59 (6.8%)
Shaking off	71 (4.0%)	12 (4.2%)	4 (3.1%)	27 (5.7%)	28 (3.2%)
Scratching	23 (1.3%)	9 (3.1%)	2 (1.5%)	6 (1.3%)	6 (0.7%)
Bodily Function					
Yawning	285 (16%)	32 (11%)	16 (12%)	103 (22%)	134 (15%)
Stretching	93 (5.3%)	8 (2.8%)	6 (4.6%)	28 (5.9%)	51 (5.8%)
Panting	771 (44%)	104 (36%)	43 (33%)	234 (49%)	390 (45%)
Trembling	5 (0.3%)	2 (0.7%)	0 (0%)	1 (0.2%)	2 (0.2%)
Regurgitating	3 (0.2%)	0 (0%)	0 (0%)	2 (0.4%)	1 (0.1%)
Eliminating	13 (0.7%)	1 (0.3%)	1 (0.8%)	2 (0.4%)	9 (1.0%)
Feces in cage	340 (19%)	48 (17%)	24 (18%)	72 (15%)	196 (22%)
Coprophagy	4 (0.2%)	1 (0.3%)	0 (0%)	0 (0%)	3 (0.3%)

Percentage of days during which behavior was displayed at least once, by length of stay and adopter preference group (long coat, small size, or ratter, herder or lap dog) for N = 289 dogs.

### Behavior change with attention

**Figure 1:**
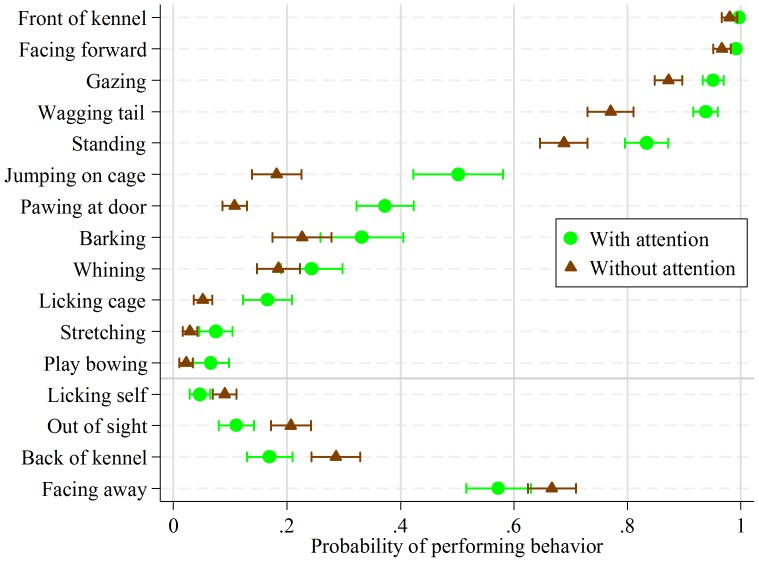
Probability of dog performing behavior, with 95% confidence intervals, by human attention type, significant at the 5% level (“with attention” signifies interactions involving two experimenters, in which one bent down and gently spoke with the dog, whereas “without attention” signifies interactions in which only one experimenter passively videotaped the dog).

We found that the following behaviors significantly increased with active attention from the experimenters: being at the front of the enclosure (OR 8.7, 95% confidence interval (CI_95%,_) 3.3–23), pawing at door (OR 4.9, CI_95%_ 3.8–6.4), jumping on door (OR 4.5, CI_95%_ 3.4–6.0), facing forward (OR 4.5, CI_95%_ 2.0–10), wagging tail (OR 4.5, CI_95%_ 3.2–6.4), licking enclosure wall or floor (OR 3.6, CI_95%_ 2.6–5.0), play bowing (OR 3.1, CI_95%_ 2.0–4.8), gazing (OR 2.9, CI_95%_ 1.9–4.3), stretching (OR 2.7, CI_95%_ 1.7–4.2), standing (OR 2.3, CI_95%_ 1.7–2.9), barking (OR 1.7, CI_95%_ 1.3–2.2), and whining (OR 1.4, CI_95%_ 1.1–1.8). The following behaviors decreased: facing away (OR 0.7, CI_95%_ 0.5–0.8), back of kennel (OR 0.5, CI_95%_ 0.4–0.7), licking themselves (OR 0.5, CI_95%_ 0.3–0.8), and being out of sight (OR 0.5, CI_95%_ 0.4–0.6). Estimated probability of dog performing these behaviors with and without attention, together with CI_95%_, are displayed in Fig. 1.

### Behavior change due to time alone

There were 131 dogs which had between 5 and 25 observations. We found that for 100 dogs (76.34%), there were no behaviors that significantly changed during the shelter stay. There were no behaviors for which more than one or two dogs changed in the same way; in other words, there was no consistency in behavioral changes. However, we could categorize dogs as becoming more active or less active. There were eight dogs who became more active and 14 who became less active (significant at the 10% level), where active was more barking, jumping, moving forward/away or less lying down. Behavior changes unrelated to activity level were, for example, less panting, more standing. We concluded, therefore, that these data do not provide evidence of significant behavioral changes of dogs during shelter stays.

### Effect of behavior on length of stay

**Figure 2:**
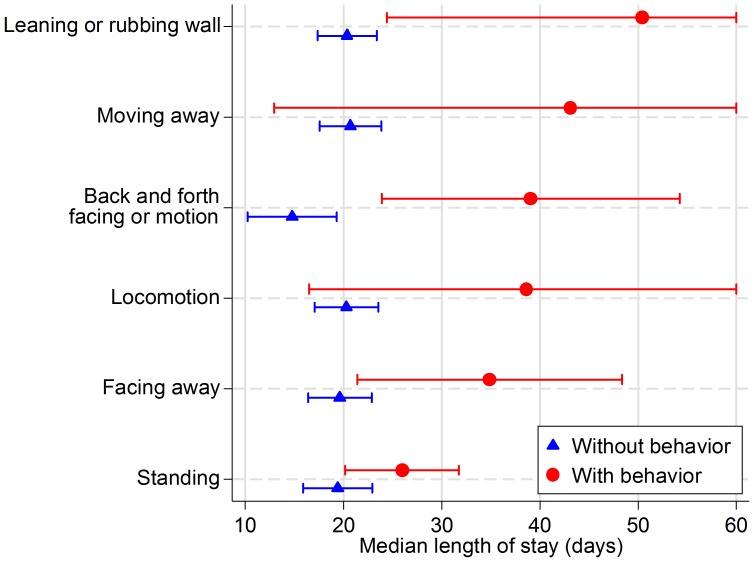
Median length of stay, by dog behavior, with 95% confidence intervals, significant at the 10% level for a short haired, medium to large, shelter dog aged over 4 months of a non- preferred breed type (i.e., not ratter, herder or lap dog).

Three behaviors were found to have a statistically significant effect on length of stay: leaning or rubbing on the enclosure wall (HR  = 0.29, P = 0.004), facing away from the front of the enclosure (HR  = 0.48, P = 0.017), and standing (HR  = 0.70, P = 0.045). Sitting was also found to have a statistically significant effect, but only for puppies (HR  = 2.04, P = 0.049). Moving away had a marginally significant effect on length of stay (HR  = 0.38, P = 0.078). Morphologically non-preferred dogs increased their median length of stay from 20 (CI_95%_ 17–23) days until adoption to 50 (CI_95%_ 24–76) days by leaning or rubbing on the enclosure wall, from 20 (CI_95%_ 16–23) to 35 (CI_95%_ 21–48) days by facing away, and from 19 (CI_95%_ 16–23) to 26 (CI_95%_ 20–32) days by standing (Fig. 2).

Combinations of behaviors were also studied: vocalization (the sum of all vocalization behaviors), locomotion (the sum of all locomotion behaviors), back and forth facing or motion (moving forward and away, facing forward and away and pacing), sitting and lying down, and rear of kennel (sum of back of kennel and out of sight). Of these, only one had a statistically significant effects on length of stay: moving or facing back and forth (HR  = 0.66, P = 0.004). Locomotion had a marginally significant effect on length of stay (HR  = 0.66, P = 0.061). These results are listed in Table 4. Morphologically non-preferred dogs increased their median length of stay from 15 to 39 days by moving and facing back and forth in the kennel, and from 20 to 38 days with increased locomotion (Fig. 2).

**Table 4:** Hazard Ratios for the Effect of Dog Behavior on Length of Stay, with P-Values and 95% Confidence Intervals, Significant at the 10% Level.

	Hazard	95% Confidence Interval		
Behavior	Ratio	Lower bound	Upper bound	p-value
Leaning or rubbing wall	0.29	0.12	0.67	0.004
Moving away	0.38	0.13	1.12	0.078
Facing away	0.48	0.26	0.88	0.017
Forward or away, facing or motion	0.66	0.50	0.88	0.004
Locomotion	0.66	0.42	1.02	0.061
Standing	0.70	0.49	0.99	0.045
Sitting^a^	2.04	1.06	3.94	0.049

Hazard ratios greater than one indicates the length of stay is increased as the behavior is increasingly performed and hazard ratios less than one indicates the length of stay is decreased as the behavior is increasingly performed.

a Sitting only reduced length of stay for dogs <4 months of age.

Human attention had no impact on the effect of behavior on length of stay (all p-values greater than 0.05); in other words, the difference in a dog's reaction to attention did not significantly affect time to adoption. As expected from the results of the earlier section, length of coat, size, and breed type had a significant effect on length of stay when included in the models in addition to behavior: having a long coat, being small sized and being a ratter, herder, or lap dog significantly shorted length of stay (p-values less than 0.01 for all three in all models). Interactions of these variables with behavior were not significant (all p-values for interaction term were greater than 0.05), meaning that being a morphologically preferred dog did not change the impact of behavior on length of stay. Once breed type, size, and coat length were accounted for, the behavior of being out of sight did not have a significant effect on length of stay (P = 0.942).

## Discussion

The results of this study showed that back and forth motion in the kennel, leaning or rubbing on the enclosure wall, standing, and facing backward significantly increase length of stay in shelter dogs. In fact, dogs that engaged in any back and forth motion in their kennel increased their length of stay by approximately 15 to 25 days. Dogs that faced away from potential adopters increased their length of stay by approximately 15 days. Dogs that engaged in enclosure contact increased their length of stay by a dramatic 30 days; whereas, standing only increased stay by approximately 5 days (Fig. 2).

Puppies improved their chances of adoption by sitting; however, puppies already have a higher adoption likelihood due to their small size and our data show that puppies are more likely to sit than juvenile or adult dogs. Therefore, teaching puppies to sit in their kennel might not be as impactful as decreasing other undesirable behavior mentioned above. Fig. 2 omits the sitting behavior, as sitting did not influence length of stay for morphologically non-preferred dogs.

Increased locomotion or hyperactivity has been reported to be problematic for owners [19]. The aversion to increased activity in the kennel observed in our data, through backward and forward motion, may parallel the aversion to increased activity in the home. Furthermore, increased activity may be perceived as stereotypic behavior indicating poor mental health or inability to cope in a confined environment.

A surprising finding was that contact with the enclosure, such as leaning on or rubbing the kennel walls, was perceived as very undesirable by adopters. Interestingly, a recent study found that leaning and rubbing on people correlated with a low sociability score on a standardized test [20]. It is possible that adopters are sensitive to these subtle behaviors that may indicate low sociability. However, an alternative hypothesis for the undesirability of these behaviors is that not the actual leaning or rubbing itself, but the passive nature of these behaviors discourages potential adopters. It is possible that adopters perceive dogs that remain stationary even when approached as uninterested, not social, and/or unhappy. This would also explain why the behavior of standing was associated with a slightly longer length of stay.

Adopters may perceive dogs that face away as unfriendly, uninterested, or fearful. However, no other fear behaviors, such as cowering, tucking of the tail, or trembling predicted a longer stay, thereby suggesting that fear responses in general do not dissuade adopters into taking a dog home. Alternatively, adopters may require the dogs to be in full sight in order to assess their morphology more fully before making a decision to adopt or not adopt. Whereas we found that being out of sight did not increase the dogs' length of stay, the failure for this behavioral variable to contribute significantly as a predictor to the model may have been in part due to the variance being accounted for by the size variable (medium to large dogs spent more time out of sight and also had a longer length of stay).

Surprisingly, adopters were not sensitive to some in-kennel behaviors previously assumed to be important [5], [6], [13]. Sitting, gazing, not barking, and not jumping on the kennel door did not significantly decrease length of stay in all dogs. In fact, jumping on adopters was reported to be a frequent behavior that shelter dogs exhibited prior to adoption [14] further suggesting that jumping does not inhibit adoption. In addition, sitting, gazing, barking, and jumping were also not predictive of adoption likelihood in out of kennel interactions [8]. These findings suggest that training shelter dogs to engage in these behaviors may be unnecessary as they do not influence adopter selection. Alternatively, some of these behaviors may be, in fact, desirable (i.e. gazing), but also appear at sufficiently high rates in an untrained shelter dog population; thus, training dogs to engage in these behaviors may not be necessary.

An additional interesting aspect of the findings is that no behaviors predicted a shorter length of stay for all dogs. This suggests that adopters were more sensitive to undesirable than to desirable behaviors. Previous literature supports such a negativity bias. People routinely place greater emphasis on negative than positive events [21].

We found that, in our population, dogs with a long coat, of a small size, and of ratter, herder, or lap dog breed type had a significantly shorter time to adoption as compared to dogs with a short coat, of medium or large size, and of other breed type. The age variable did not contribute significantly to the model, as almost all puppies were also of small size. We also found that color and mode of intake did not predict length of stay: light colored and owner surrendered dogs did not have a shorter length of stay as compared to darker colored or stray or confiscated dogs. Brown et al. [16] report that, out of the previous studies that assessed the effect of color on adoption likelihood, only four out of eight reported a statistically significant effect. In addition, to the best of the authors' knowledge, only two studies have reported a statistically significant effect of mode of intake, one of which relied on questionnaire data [6], [13].

Our data showed some behavioral differences between dogs of different size. Medium and large dogs were more likely to be out of sight, in the back of the kennel, facing away from the observer, moving back and forward in the kennel, panting, whining, and stretching. It is possible that larger dogs were more sensitive to the outside temperatures and, thus, sought shade in the back of the kennel, which is further supported by the finding that larger dogs spent more time panting.

No theoretically significant behavioral differences existed between dogs of different breed type. However, the “preferred” breed types (i.e., ratters, herders, and lap dogs) as a group were more likely to whine and less likely to be in a lying down position. Additionally, long-haired dogs (which were included in the morphologically preferred category) were more likely to be out of sight, in the back of the kennel, facing away from the observer, and moving back and forward in the kennel. The lack of behavioral differences between breeds may be partially explained by lack of purebred dogs in the shelter population. It is possible (even probable) that the dog breed labels were inaccurate [22]. Furthermore, even in purebred dogs, behavioral differences within breeds may be quite large (for a review see [23]).

A surprising finding was that, when assessed for individual change in behavior over time, no systematic patterns emerged. Our results, in addition to the previous research [11], [12], [19], suggest that different dogs may respond differently to prolonged confinement with some exhibiting less activity and some more.

In order to assess the reactions of dogs to different kinds and numbers of visitors, we looked at the changes in dog behavior when approached by a single passive experimenter and by a pair of experimenters, one of whom verbally engaged with the dog. We found dogs mirrored the experimenter's engagement by attending more to the experimenters (spending more time at the front of the enclosure, less time in the back of the kennel and out of sight, facing forward, and gazing) as well as being more active in their interactions (pawing at the cage door, jumping on the cage door, standing, wagging the tail, play bowing, whining, and barking). Furthermore, dogs spent more time licking the enclosure wall or floor and stretching when approached by the pair of experimenters as opposed to the single passive experimenter. Our results are in accordance with previous research, which has found that dogs in general behave appropriately to friendly greetings by strangers [24].

It remains unclear whether adopters that come in pairs or groups and are more active in interacting with dogs elicit more appropriate behavior. Whereas an increase in time spent at the front of the enclosure was desirable, we found that an increase in time spent standing was undesirable. Furthermore, we found an increase in barking when experimenters were more actively interacting with the dogs. Although barking did not influence length of stay in our study, this behavior may be undesirable for other reasons such as increasing the overall noise of the shelter, which has been found to be a stressor for dogs [25], [26]. All experimenters in the current study were female; thus, we could not assess the behavior of dogs to different genders of adopters. However, previous research suggests an interaction between dog sex and human gender [27], [28]. Future studies may choose to explore this interaction further as well as explore whether dogs' in-kennel behavior influences women and men adopters differently.

Our findings may be used for the development of targeted behavioral interventions to increase adoptions in shelter dogs. By subdividing dogs into morphologically preferred and non-preferred groups and concentrating scarce resources on behavioral training for morphologically non-preferred dogs due to their prolonged stay, shelters may target the specific behaviors that influence adopters and the dogs that need most assistance in finding homes. Furthermore, these findings will allow shelters to save resources by focusing behavioral modification efforts only on behaviors that have been validated as salient to adopters.

A limitation of the present study is that the in-kennel behavior of dogs was recorded by observers who may differ from adopters in significant ways, thereby eliciting different behaviors from the dogs. Whereas we attempted to control for this limitation by recording behavior with a small and inconspicuous hand-held video camera, wearing casual clothes, recording only during the shelter's open hours for just 1 min at a time, and varying the identify and number of observers, there still remains a possibility that the dogs were able to discriminate between experimenters and true adopters.

Caution must be taken when interpreting this correlational data. It would be incorrect to assume a causal relationship between behavior and length of stay from the present results. Future research should examine whether such a causal relationship exists by experimentally modifying dogs' in-kennel behavior and evaluating its effect on adopters.

Furthermore, it is possible that our findings may be specific to the animal shelter at which data was collected and may not be generalizable to other populations. Shelters may differ in the population of dogs that are offered for adoption, in the staff that make decisions at the shelter, and populations of adopters (demographic information of adopters at ACAS is reported in Protopopova and Wynne [8],). Therefore, multi-site evaluations are recommended to evaluate the generalizability of these results.

## Conclusion

Our findings suggest that dogs' in-kennel behavior predicts time to adoption. Dogs that exhibited increased back and forth motion in the kennel, contact with the enclosure, and faced away, regardless of morphology, had a longer length of stay at the shelter. Behaviors previously considered important, such as sitting, gazing, not barking, and not jumping on the kennel door did not predict length of stay in all dogs. No behaviors changed systematically with time. These findings may allow shelters to save resources by focusing behavioral modification efforts only on behaviors likely to influence adopters' choices, and will lead to targeted and effective shelter interventions to increase adoption rates in shelter dogs.
